# Electrophysiological Correlates of Binocular Stereo Depth without Binocular Disparities

**DOI:** 10.1371/journal.pone.0040562

**Published:** 2012-08-02

**Authors:** Karoline Spang, Barbara Gillam, Manfred Fahle

**Affiliations:** 1 Centre for Cognitive Science, University of Bremen, Bremen, Germany; 2 School of Psychology, University of New South Wales, Sydney, Australia; 3 The Henry Wellcome Laboratories for Vision Science, City University, London, United Kingdom; University of Muenster, Germany

## Abstract

A small region of background presented to only one eye in an otherwise binocular display may, under certain conditions, be resolved in the visual system by interpreting the region as a small gap between two similar objects placed at different depths, with the gap hidden in one eye by parallax. This has been called monocular gap stereopsis. We investigated the electrophysiological correlate of this type of stereopsis by means of sum potential recordings in 12 observers, comparing VEP's for this stimulus (“Gillam Stereo”, Author BG has strong reservations about this term) with those for similar stimuli containing disparity based depth and with no depth (flat). In addition we included several control stimuli. The results show a pronounced early negative potential at a latency of around 170 ms (N170) for all stimuli containing non- identical elements, be they a difference caused by binocular disparity or by completely unmatched monocular contours. A second negative potential with latency around 270 ms (N270), on the other hand, is present only with stimuli leading to fusion and the perception of depth. This second component is similar for disparity-based stereopsis and monocular gap, or “Gillam Stereo” although slightly more pronounced for the former. We conjecture that the first component is related to the detection of differences between the images of the two eyes that may then either be fused, leading to stereopsis and the corresponding second potential, or else to inhibition and rivalry without a later trace in the VEP. The finding that that “Gillam Stereo” leads to cortical responses at the same short latencies as disparity based stereopsis indicates that it may partly rely on quite early cortical mechanisms.

## Introduction

**Figure 1:**
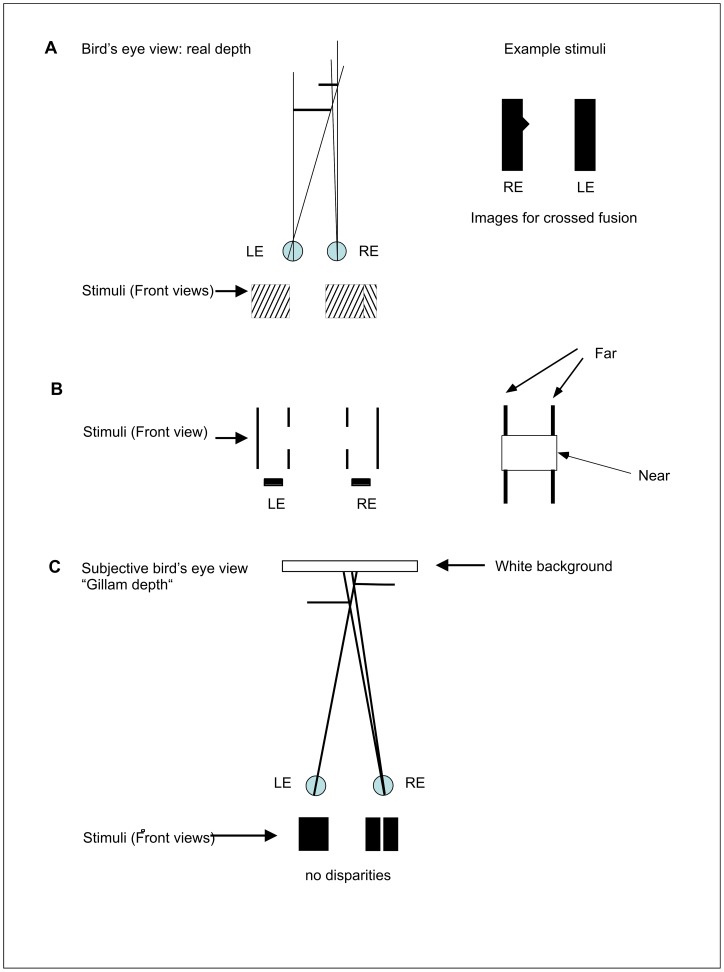
A–C Examples of stimuli mentioned in the introduction. A Da Vinci stereopsis. B Phantom stereopsis. C “Gillam Stereo”. A. Da Vinci stereopsis is defined as the perception of a monocular surface in depth relative to a proximal binocular surface. This can occur when the monocular surface is in a valid location for its occlusion in the other eye by the binocular surface. A bird's eye view of this situation showing the basic spatial layout is displayed on the upper left. On the right is an example of Da Vinci stereopsis where the monocular triangular shape is seen in depth after crossed fusion. The resulting percept is of a rectangle with a small triangle floating behind the rectangle on its right side. B. Phantom stereopsis. In this stereogram (front view displayed on the left) the central part of the left black vertical line can be seen only by the left eye (LE) and the central part of the right vertical line only by the right eye (RE). Only a nearer surface hiding different parts of the images for the two eyes would account for these monocular image components and that is what is seen in the form of a “phantom” surface nearer than the lines as is illustrated in the sketch on the right (compare also Kanizsàs triangle). C. “Gillam Stereo”: Two black squares at different depths are placed so that one eye can see between them whereas the other eye cannot. The images in this case can be simulated by a stereogram as shown at the bottom of Figure 1c. One eye's image has a monocular gap that has no corresponding image in the other eye. Ecologically such a stimulus could only occur for two objects separated in depth at the gap and this is what is seen despite a lack of binocular disparity at the depth step. Fusion of the stereogram elicits perception of two squares separated in depth with the left one closer (with cross fusion the right is closer).

In recent years it has become clear that binocular depth perception is not restricted to locations in the perceived environment with features that can be explicitly matched in the two eyes. Depth can also be recovered for features that are imaged only monocularly if these are placed in an informative binocular context. We do not refer here to what are classically called monocular depth cues. The depth perception we refer to is entirely binocular in origin but incorporates monocular (unpaired) features. [For reviews see Harris & Wilcox [1], and Gillam [2], There are three major categories of such effects investigated so far. The first is the depth seen for an unpaired feature placed in the “occlusion zone” of a binocular surface (Fig. 1A, [3], [4]. This is also known as “da Vinci stereopsis” since Leonardo da Vinci first pointed out that occluding surfaces may hide more distant surfaces differentially for the two eyes.

The second phenomenon was also first described by Nakayama & Shimojo [3]. In this case the presence of monocular features in a context of fused features elicits the impression of a phantom occluding surface (bounded by subjective contours) that “accounts for” the monocularity of such features (Fig. 1B). The most striking of these phantom surfaces are those produced by unpaired vertical elements first described by Anderson [5]–[7] and the “phantom rectangle” investigated by Gillam and Nakayama and others [8]–[10]. We call this class of phenomena “phantom stereopsis” [2].

The third class of depth resulting from an unpaired region (and the one with which this paper is concerned) is known as “monocular gap stereopsis” [11] or “unpaired background stereopsis” [12] and has been extensively investigated by Gillam and colleagues [11]–[14]. This phenomenon is different from the two classes described previously in that the monocular feature is not itself seen in depth. Nor does it elicit a phantom surface (Fig. 1A, 2A). Monocular gap stereopsis mimics an ecological situation in which two side by side frontal plane rectangles of a solid color are placed at different depths so that one eye can see the background between them and the other eye cannot. The only binocular disparity present in this case is at the outer edges of the two rectangles. Using stereograms it can be demonstrated that the visual system can use the monocular gap to resolve this situation. The presence of a vertical gap in one of two otherwise fusible binocular solid rectangles with a disparity only at their outer edges causes the fused surface to split binocularly at the gap into two surfaces at different depths (see Figure 1C).

**Figure 2:**
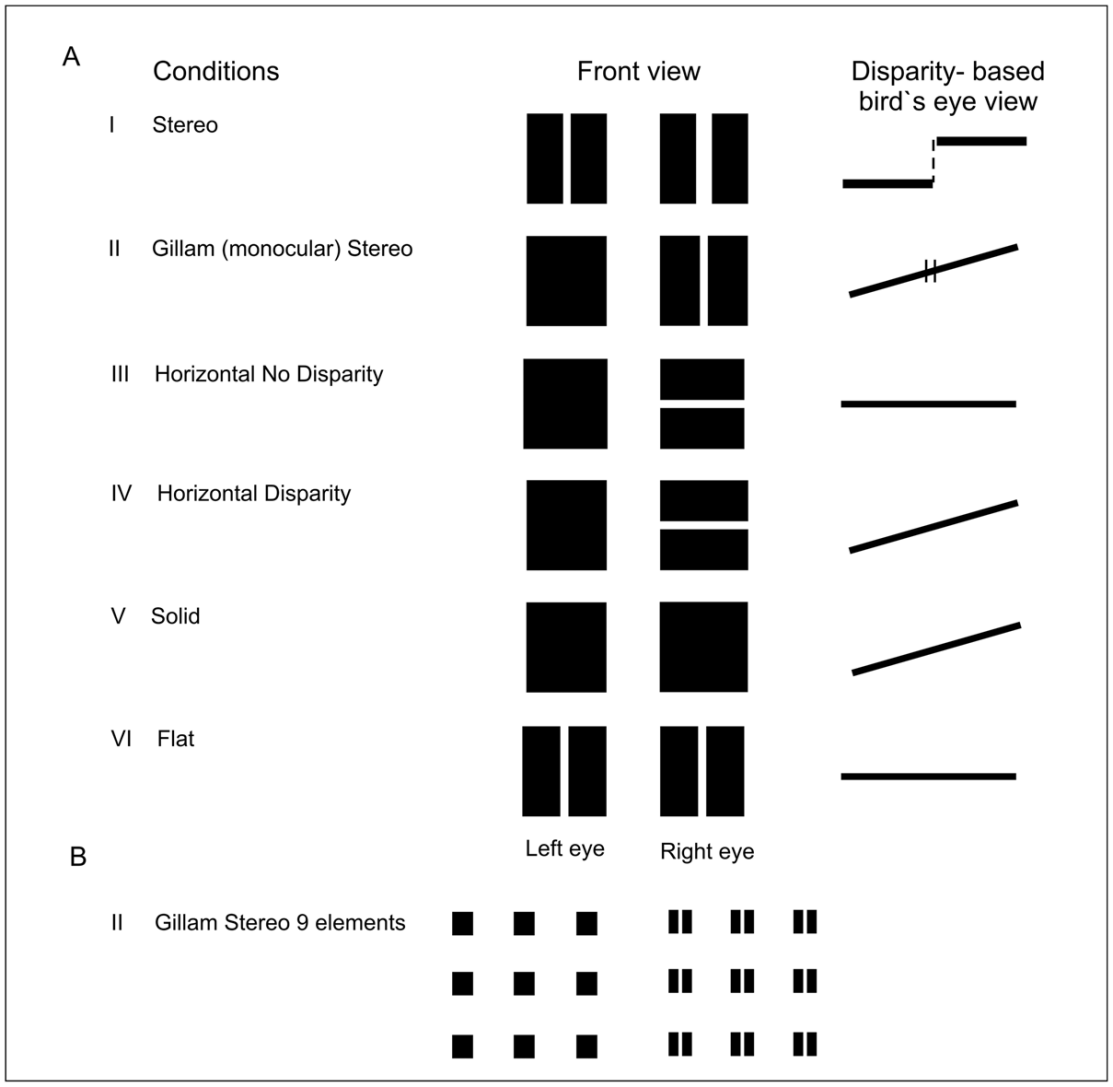
A, B Schematics of the stimuli used in the study. The “Front view” shows the stimuli in two columns. Stimuli in the left column are presented to one eye and those in the right column to the other eye. The bird`s eye views represent reconstructions of the depth relationships predicted from binocular horizontal disparity alone. The following stimuli were used: I “Stereo”, in which depth is based on horizontal disparity present at all vertical contours; II “Gillam Stereo” Depth based on a monocular vertical gap with horizontal disparities present only at the outer edges. (Hence, the disparity- based display differs from the “Stereo” condition, while the subjective impression does not.) III “Horizontal No Disparity” A condition with a monocular horizontal gap with no horizontal disparities; IV “Horizontal Disparity” As III but with horizontal disparities at the outer edges; V “Solid” A condition with neither horizontal nor vertical gaps, but with outer edge disparities; VI “Flat” A condition with a binocular vertical gap without any disparities. Please note that stimuli I and II are seen in depth while the others are seen as flat (V, VI) or even rivalrous (III, IV). The slant predicted in conditions IV and V by the use of disparity at the outer edges (see bird`s eye view) is barely noticed by the observers. If crossed fusion of the stimuli is used the depths will reverse (when there is depth). Figure 2B shows an example of the displays with 9 elements (here with the Gillam Stereo stimulus, II). The other conditions follow the same spatial layout.

In all cases involving monocular regions, depth is ambiguous although the geometry always specifies a minimum depth. For details see [3], [4], [8], [11]. Monocular gap stereopsis, more than any of the other cases in which depth is elicited by unpaired regions, gives metric depth (increasing with gap width) which satisfies the minimum depth constraint for that stimulus and is reliable across observers. Monocular stereopsis may even utilise the same mechanisms as regular stereopsis [14]. It is easy to create comparable regular stereo and monocular gap stereo stimuli by making one set of rectangles in which there is a binocular gap with a width disparity and another set with a monocular gap (see Figure 2A) whose width is equal to the disparity in the other set. The gap in the latter case can be treated as a disparity if the solid image in the other eye is treated by the visual system as two adjacent rectangles with zero separation. This does appear to be the case in that the depth obtained as a function of disparity in the stimulus with the binocular gap has the same magnitude as the depth obtained as a function of the monocular gap in the second case [11], [13]. Furthermore Pianta & Gillam [14] found very similar thresholds for depth in the two cases as a function of stimulus duration and also cross adaptation between the two forms of stereopsis. In the stimuli used in their studies the width of the gap was the same as the disparity at the outer vertical edges of the figure. This support may account for the strong metric depth obtained. However Pianta and Gillam [13] showed that the apparent depth difference of the rectangles at the gap was not simply a matter of transporting a depth signal from the outer edges to the gap. When solid rectangles with no gap were used, the thresholds for detection of depth were far higher than those obtained for the monocular gap stimulus and there was no cross adaptation with regular stereopsis. This reflects the well-known impoverishment of perceived depth in response to rectangular stimuli with a width disparity [15]. (It has been found that even when the outer edge disparity is eliminated so that the overall widths of the figures in the two eyes are the same, depth is still seen at the gap and the resulting two rectangles look slanted as required by the geometry of this stimulus [13]. This situation can produce greater individual differences in the way the geometry is resolved into a combination of depth and slant than the more reliable case we used in these studies where no slant is required in the stereo solution.).

The present studies were devised to explore electrophysiologically the similarity or otherwise of the response of early visual channels to regular stereopsis and monocular gap stereopsis (“Gillam Stereo”). We used monocular gap stereopsis for three reasons. First it is very easy as indicated above to create stimuli for paired and unpaired stereopsis that are very similar. In the one case the gap is binocular with a given disparity and in the other it is monocular with a width equal to the same given disparity. Secondly these two stimuli have been shown to produce the same depth magnitude as a function of gap/disparity. Thirdly there is psychophysical literature, described above, showing a strong similarity between the nature of the depth response in these two cases.

Only a few studies so far have investigated the electrophysiological correlates of stereoscopic depth perception [16]–[20]. Our main aim was to discriminate between basically two different sources for the perception of depth in these types of stimuli. The first one – favoured by us on the basis of the psychophysical results of earlier studies comparing monocular gap and disparity-based stereopsis – postulates a relatively early location of this type of depth perception during cortical processing. An alternative explanation would rely on more cognitive mechanisms, based on rather sophisticated knowledge about the visual world. The hypothesis of an early origin of monocular gap stereopsis would require evoked potentials similar to the ones evoked by disparity-based depth already over the occipital cortex, while the more cognitive hypothesis would not. We did indeed find such relatively early potentials, most prominently over the occipital skull, i.e. early visual cortices.

## Materials and Methods

### Ethics Statement

The study was approved by the ethics committee at the University of Bremen and done in full compliance with the guidelines of this committee. The tenets of the Declaration of Helsinki were strictly observed.

### Observers

In a pilot study, seven observers participated in tests with a subset of the stimuli employed in the study reported here. Twelve observers participated in the study proper. All observers gave their informed consent to participate in the study. They were naïve concerning the exact problem investigated in the study. Data of one additional observer had to be discarded due to extensive eye-blinks. All but two subjects were students of Bremen University and were aged between 21 and 51 years (mean: 27 years ±10 se) and were mostly female (11).

All observers had normal or corrected-to-be-normal acuity and normal stereo vision as tested by the TNO -Test (i.e. better than 40 arcseconds). In a preliminary practice session the conditions were presented to the subjects in random order and subjects had to indicate whether or not the stimulus was slanted in depth and which side of the stimulus – right or left – was “protruding” from the monitor. All participants easily solved this task.

### Stimuli and set-up

All stimuli were presented on an EIZO T662-T CRT under PC control with a resolution of 1280×980 pixels at 144 Hz (monocular frame rate 72 Hz) in a dimmed room. The high temporal resolution was achieved through special hardware inserting additional lines and thus converting each half of a conventional frame into a full frame. Liquid crystal diode (LCD) shutter goggles (CrystalEyes® Model CE-1) synchronised to the monitor ensured that each of the eyes saw only the upper or else the lower half of each original frame, each blown up to a full frame, with the two frames superimposed upon each other.

The stimuli displayed consisted of dark rectangles with a side length of around 1.2 arcdeg and a height of 1.5 arcdeg viewed at a distance of 140 cm. The luminance of the background was around 74 cd/m^2^ and that of the stimuli proper around 2.4 cd/m^2^. The shutter goggles attenuated luminance by a factor of approximately 2 when open and more than 20 when closed.

The following stimuli were employed (Fig. 2A).

(I) A rectangle for which both eyes' images had a central vertical gap. In one eye this gap was 8 arcmin wide and in the other eye 16 arcmin so that there was a disparity of 8 arcmin or 2 times 4 arcmin in different directions with the same disparities at the outer edges. These disparities resulted in the percept of two flat rectangles, separated in depth. We refer to this condition as Stereo, or Disparity Based Stereo.

(II) A rectangle for which one eye's image had a vertical gap whose width equalled that of the disparity in (I) (8 arcmin.), while the image in the other eye was a solid rectangle. The outer edges of the rectangle images had a disparity equal to the gap width. (The image with the gap was the wider one). We refer to this condition as “Gillam Stereo”.

(III, IV) A rectangle for which one eye's image had a horizontal gap, while the image in the other eye was a solid rectangle. In case (III) (“Horizontal gap no disparity”) the images had no outer edge disparity whereas in case (IV) (“Horizontal gap disparity”) there was an outer edge disparity equal to that in (I) and (II).

(V) A solid rectangle in both eyes without any “internal” gaps but with disparity at the outer edges (“Solid”)).

(VI) A rectangle with a central vertical gap in both eyes (as in I), but without any disparity at the gap or at the outer edges (“Flat”).

For all the above stimuli (except for VI, where the images were identical) 50% of the trials had the wider image and/or gap in the left eye and 50% in the right eye. There were two versions of all the stimulus types. In the first version, a single stimulus element was displayed while in the second version nine identical elements were displayed as a 3*3 matrix with an inter-element distance of 4 arcdeg (Fig. 2B). The nine element stimuli were expected to yield larger amplitudes of evoked potentials than the single stimuli [21].

Stimuli were displayed for 1000 ms, with a homogeneous grey background present for 500 ms between presentations. Observers were asked to fixate the stimulus and to try to decide in which direction – if any- the stimulus was slanted in depth, but without reacting to the stimulus (no response required). The image that had the wider gap (disparity conditions) or the monocular gap (monocular gap conditions) varied pseudo-randomly across presentations. In the monocular case, this meant that the vertical or horizontal gap (see Fig. 2A) appeared randomly in the right or else left eye within each session.

The different types of stimuli were presented in pseudo random order in sessions with 480 stimulus-sweeps (80 sweeps for each of the 6 conditions, over-all duration ∼ 12 min). We recorded 2 sessions for each stimulus version (1 element and 9 elements), sampling 160 sweeps for each condition. Total recording time was ∼ 48 min for each participant.

### Electrophysiological recording

We recorded from five positions on the scalp (O_1_, O_2_, P_z_ T_5_, T_6,_) by means of gold-cup electrodes according to the international 10–20 system, referenced to F_z_. Potentials of O_1_ and O_2_ were subsequently pooled to produce O_t_, resulting in datasets for four electrode sites. Electrode impedance was below 10 kΩ. Blinks were monitored by vertical electrooculogram with electrodes placed above and below the right eye and data containing blinks were excluded. Potentials were amplified by Toennies physiological amplifiers, band-pass filtered (from 0.5 to 130 Hz with an additional notch filter at 50 Hz) and subsequently digitized at a sampling frequency of 400 Hz. Data were fed into a PC by means of an A/D converter card (Data Translation, Inc) and custom-made software.

### Data analysis: Averaging & Differentiation

Data analysis was performed with software developed in house based on IGOR Pro software (version 4.0, WaveMetrics, Inc., Oregon). Single subject data were averaged separately for each condition and electrode location. The averaging period lasted 1000 ms per stimulus sweep starting with a temporal offset of 200 ms before stimulus presentation. Automated artefact rejection was applied to eliminate data epochs contaminated by blinks exceeding ±100 µV amplitude. Usually, less than 15% of sweeps had to be rejected.

### Data analysis: Statistics

**Figure 3:**
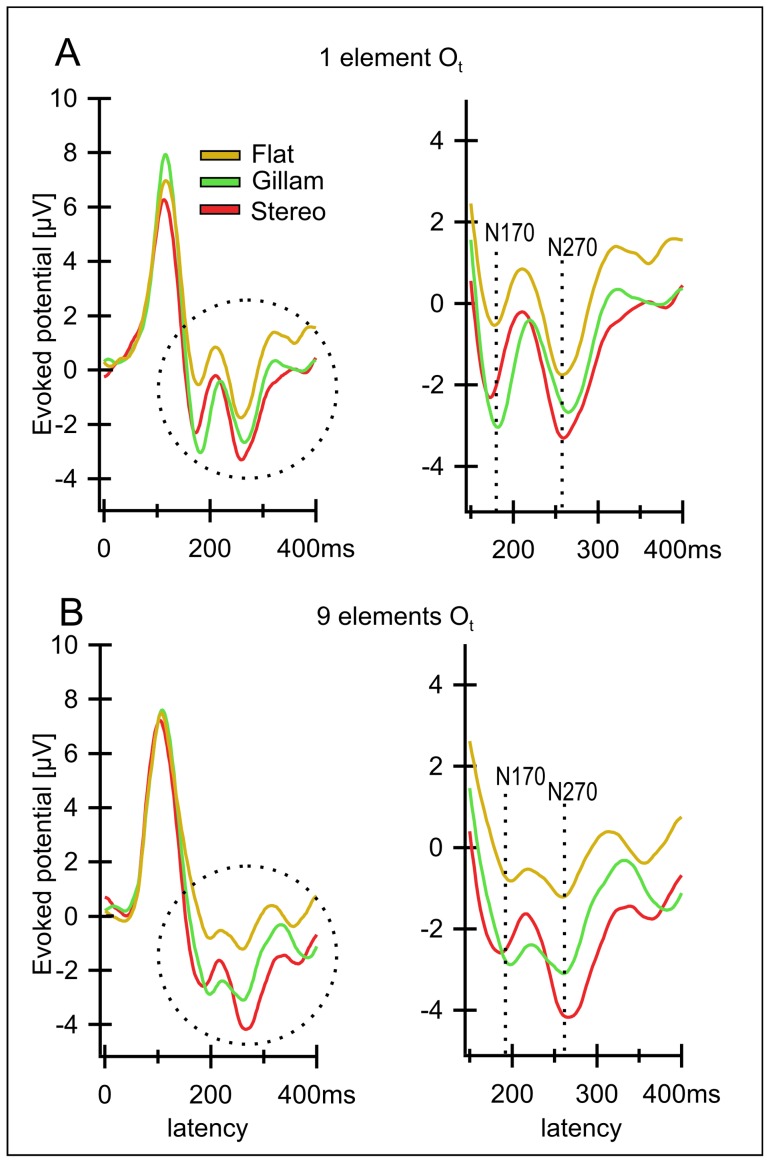
A, B Grand averages for electrode O_t_ (average of O_1_ and O_2_) over all 12 observers for three conditions. A) Results for the one element version. B) Version with 9 elements yields relatively similar results. The conditions displayed are: “Disparity Based Stereo (condition I, red lines), Gillam Stereo (II, green lines) and Flat (VI, yellow lines)”. Right column of figure shows a magnification of the areas marked with a circle in the left column. There are two distinct peak negativities at latencies of 170 ms (N170) and 270 ms (N270) that are similar in the two conditions containing depth (Stereo, Gillam) separated by a small positive peak at a latency of around 200 ms. In the flat condition (VI, yellow line), containing no depth, the two negativities are smaller (one element version) or almost missing (9 elements version).

**Figure 4:**
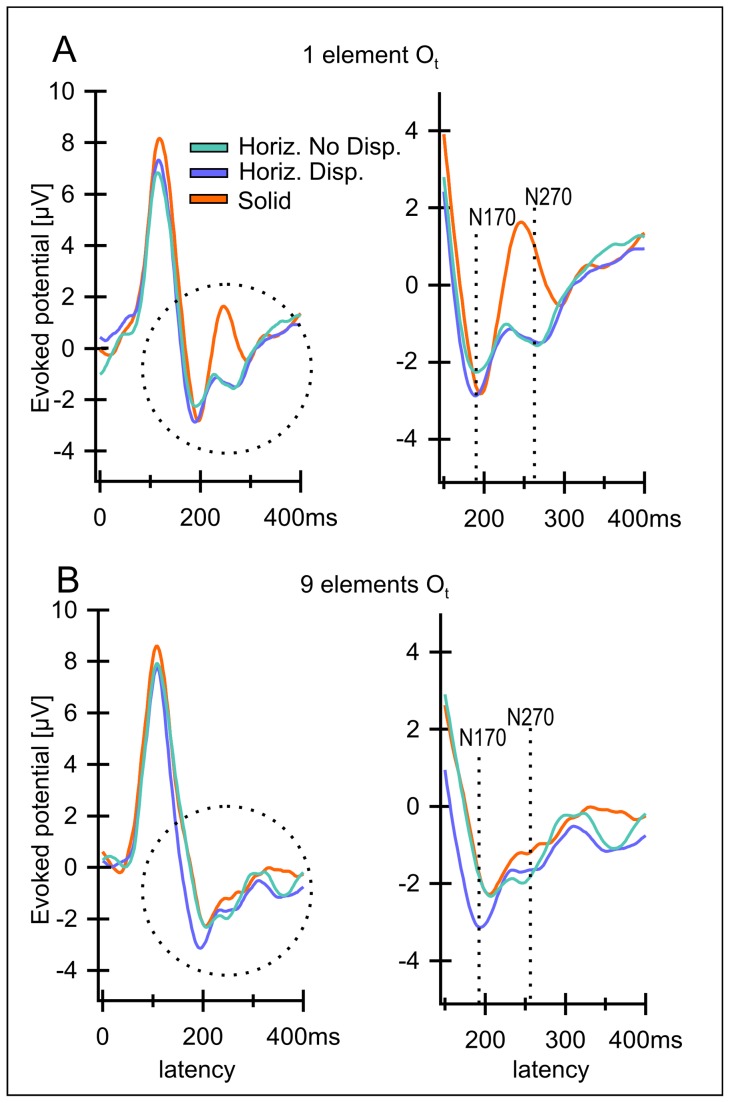
A, B Grand averages for electrode O_t_ over all 12 observers for the three control conditions. A) One element version, B) 9 element version. Right column shows a magnification of the areas marked with a circle. The conditions were “Horizontal No Disparity” (III, light blue lines), “Horizontal Disparity” (IV, dark blue lines) and “Solid” (V, orange lines)”. The N170 is almost as strong for all 3 conditions as for the stimuli containing depth (compare Figure 3). The N270 is missing in the case of the “Solid” stimulus (V) and very small for the conditions “Horizontal No Disparity” (III) and Horizontal Disparity (IV) differing significantly from the conditions containing depth (compare Figure 3).

We employed different types of statistics. In the more conventional type, which seems most adequate for the type of potentials plotted in Figs. 3 & 4, we identified the two positive extremes of the response curves occurring between 60 and 140 ms after stimulus onset (P100), and between 160 und 230 ms (P200) as well as the two negative peaks between 130 and 200 ms (N170) and between 210 and 300 ms (N 270) in each observer. We chose the negative peaks for further analysis since we found a significant influence of stereo depth on these potentials in an earlier study [19]. For the amplitudes of N 170 and N 270 limited to O_t_, i.e. the mean of the values of the electrodes O1 and O2, we calculated a one-factor repeated measure ANOVA (factor stimulus, 6 levels) for both stimulus versions (1 element, 9 elements) using SPSS 12.0 (SPSS Inc., Chicago). If necessary, p-values were adjusted by Greenhouse–Geisser corrections.

As a second statistical measure, we calculated the means of all observers (grand averages) for the evoked potentials over a 400 ms period, starting with stimulus presentation, as well as confidence intervals of the means for p = 0.05 and plotted these means for all conditions.

A last and least conventional statistical measure consisted of averaging potentials (or differences between potentials) for two relevant time intervals, 170±12.5 ms as well as 265±12.5 ms after stimulus onset – corresponding to the negative peaks in the grand averages of our data – by means of a repeated measure one-factor ANOVA also limited to O_t_ as well as by means of paired t-tests for pre selected conditions. We expected more negative potentials for the stimuli eliciting depth than for the flat stimuli; and only differences between the conditions after about 100 ms. We were not interested in attention-driven effects with latencies above 300 ms [19].

### Data analysis: Wavelet analysis

Apart from the conventional analysis of latencies and amplitudes of evoked responses we performed a wavelet analysis of the data and plotted and compared the power spectra of the relevant conditions.

### Time-frequency analysis

A Morlet based wavelet transform with a width of 6 cycles was used for the inspection of power changes within defined frequency bands (4–80 Hz). The core routine was provided by Torrence and Compo [22]. Trials with artefacts identified in the ERP analysis were not included in the time-frequency analysis, which was computed over a time span between −200 and 800 ms relative to stimulus onset to avoid border artefacts. In contrast to the ERP analysis, the data were not filtered. We investigated normalized median power values of total activity (evoked and induced – for details see [23]. The procedure was as follows: For each subject separately, we computed the power values (in µV^2^) in each trial for each frequency (*f*) and summarized all trials by taking the median power over all trials at each frequency and point in time. Moreover, we normalized the power value (*P_f_*) at each time point *t* by the mean of the baseline power (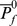) according to 


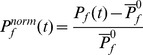


Therefore the resulting normalized power value (*P ^norm^_f_* ) at time *t* has no unit and represents the activity relative to baseline. As baseline we used the time window 500 ms prior to stimulus onset. Since the normalization factor (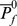) is frequency-dependent, the normalization also accounts for the fact that high frequencies have less power in the EEG than low frequencies. Hence, the normalized power values represent the frequency-specific relative increase compared to baseline power. Finally, the normalized data were averaged over all subjects yielding means and standard errors of the means. We obtained six time-frequency plots for each electrode for the six experimental stimuli used (this was calculated both for the version with one element and the one with nine elements). We then averaged the power within each of four frequency bands (4–8 Hz  =  *θ*; 8–12 Hz  =  *α*; 12–30  =  *β*; 30–80 Hz  =  *γ*) plotted the results for each frequency band of the relevant conditions as a function of time (latency) which allowed us to compare different conditions.

### Statistics

Statistical analysis was performed using SPSS 12.0 (SPSS Inc. Chicago). All results were validated by using repeated measurement ANOVAs. Wherever appropriate, p-values were adjusted by Greenhouse–Geisser corrections. Pairwise comparisons were conducted by using post-hoc t-tests. The correlations computed in this study report Pearson's correlation coefficients. Only the power in the slow wave (θ band) data for O_t,_ i.e. the mean of O1 and O2, were statistically evaluated because we expected the biggest differences in this band by inspection. For each condition and for the two versions the maximum amplitude of the power spectrum and the latency were found. The amplitudes were normalized by subtracting at this maximum the mean for all conditions from the individual value.

## Results

The statistical analysis produced significant main effects for all experiments but generally in line with the expected differences (see Tables 1–3 for details). The results for the contrast between stereo versus non- stereo conditions on the one hand and the confounds on the other hand will be discussed separately below.

**Table 1:** Two-tailed pair wise comparisons of results for “Stereo”, “Gillam” and “Flat” conditions based on the amplitude of the N 170 peak as well as for the mean potential in the time interval T170±12.5 ms for both 1 and 9 element stimuli for the combined occipital electrodes (O_t_).

	version with one element		version with nine elements	
	**N170**	**T170±12.5**	**N170**	**T170±12.5**
Stereo (I) vs. Flat (VI)	**	**	**	***
Gillam (II) vs. Flat (VI)	***	**	**	**
Gillam (II) vs. Stereo (I)	Trend	Ø	Ø	Ø

These tables contain 36 pairwise comparisons defined a priori. Note, that on statistical grounds one would expect in random data: 4 trends (p<0.1), 2 significant differences (p<0.05), 0.4 highly significant differences (p<0.01) and 0.04 differences (i.e. 1 in 200 tables) significant at p<0.001.

Significances are marked with *** for p<.001, ** for p<.01, * for p<.05, trend <1.

**Table 2:** One-tailed pair wise comparisons of results for “Stereo”, “Gillam” and “Flat” conditions based on the amplitude of the N 270 peak as well as for the mean potential in the time interval T265±12.5 ms for both 1 and 9 element stimuli.

	version with one element		version with nine elements	
	**N270**	**T265±12.5**	**N270**	**T265±12.5**
Stereo (I) vs. Flat (VI)	**	**	**	**
Gillam (II) vs. Flat (VI)	***	*	Trend	*
Gillam (II) vs. Stereo (I)	Ø	Ø	*	*

Significances are marked with *** for p<.001, ** for p<.01, * for p<.05, trend <1.

**Table 3:** One-tailed pair wise comparisons of results for “Gillam”, “Horizontal” with and without Disparity at the outer edges and “Solid” conditions based on the amplitude of the N 270 peak as well as for the mean potential in the time interval T265±12.5 ms for both 1 and 9 element stimuli.

	version with one element		version with nine elements	
	**N270**	**T265±12.5**	**N270**	**T265±12.5**
Gillam (II) vs. Horizontal Disp (IV)	*	*	Ø	*
Gillam (II) vs. Solid (V)	**	**	Trend	*
Gillam (II) vs. Horizontal No Disp (III)	*	*	Trend	*

Significances are marked with *** for p<.001, ** for p<.01, * for p<.05, trend <1.

### “Disparity Based Stereo” versus “Gillam- Stereo”

The central question we addressed is whether or not perceived depth based on a monocular gap would evoke similar cortical potentials at short latencies as depth based on disparity. Earlier studies found disparity related potentials [16], [18]–[20] but we had to reproduce these effects for our stimuli. We were specifically interested in comparing these potentials over the occipital pole, since similar potentials there at short latencies would be an argument for a rather “early” source of monocular gap stereo in the brain. To that end, we started by comparing three different types of stimuli that did or did not contain disparity cues. These were the stimulus types I (“Disparity Based Stereo”), II (“Gillam Stereo”, i.e. depth without disparity) and VI (“Flat”, i.e. no depth at all). As indicated in the Methods section, all stimulus conditions were measured in two versions, the first containing a single element while the second version contained nine elements (Fig. 2B). The resulting grand average potentials over all observers for electrode O_t_ are shown in Figures 3A, B. Inspection of the curves shows two distinct negativities, one at roughly 170 ms (N170) and another one at 270 ms (N270) separated by a relatively small positivity with a latency around 200 ms (P200). The amplitudes of these two negative potentials are most prominent over the occipital cortex, the electrode sites O_1_ and O_2._ Results for the potentials evoked in the disparity-based stereo condition are quite similar to those of the Gillam condition while both differ markedly from those in the flat condition, especially for 9 elements (Fig. 3B). More precisely, the N 170 and N 270 peaks for the stereo condition (I) as well as for the Gillam condition (II) are clearly more pronounced than for the flat condition (VI). The N 170 for “Gillam Stereo” (II) is even more pronounced than for the stereo condition while the opposite is true for the N 270. When 9 elements are presented the difference relative to the stimulus without depth is slightly higher for the Gillam stimulus at peak N 170 compared to the classical stereo condition while slightly less pronounced for the N270. Fig. 3B right side gives a clear impression of the relevant part of the curve. Please note that in order to show the difference more clearly the first 150 ms are not displayed and therefore the scale is different to Figure 3B left side. All in all, the “Gillam Stereo” stimulus elicits a very similar form as well as time course as the disparity-based depth stimulus. This visual impression is supported by an ANOVA (see Table 1 & 2).

As we pointed out in the introduction, the two stereo stimuli (I, II) and the flat stimulus (VI) served to test the difference between stimuli eliciting depth and those that do not. (Because these meaningful pair wise comparisons were chosen before the experiment based on theoretical considerations we deemed it appropriate to abstain from a correction for multiple comparisons; see also legend of Table 1).

Tables 1 and 2 show comparisons of disparity based and “Gillam Stereo” with the flat condition and with each other for the 170 and 270 peaks (and intervals of +/−12.5 ms around each peak) respectively. The N170 (Table 1) peaks and intervals of both the disparity and the Gillam condition differ significantly from those of the flat condition, both for the 1 element and the 9 element conditions. The N270 (Table 2) peaks and intervals also show significant differences between the two forms of stereo (“Disparity Based” and “Gillam”) and the “Flat” condition except for the N270 peak for 9 elements where the “Gillam” versus “Flat” comparison yielded only a trend. Peaks and intervals for “Gillam Stereo” and “Disparity Based Stereo” differed significantly from each other only for the N270 nine-element-condition.

Figure 4 plots the corresponding results for the remaining three conditions: “Horizontal” (No Disparity, III), “Horizontal Disparity” (IV) and “Solid” (V), none of which produces a clear impression of depth (since disparities at the outer edges do not usually produce an impression of depth, see e.g. McKee [15]). None of the three conditions yields the clear N270 that was present in the depth inducing conditions, while they did elicit N170 potentials. Please note that the N270 for conditions III (no disparity at outer edges) and IV (disparity at outer edges) are virtually identical, their difference being not significant (as demonstrated in subsequent figures) reflecting, in our opinion, the irrelevance of these peripheral contours for the evoked response and for the perception of depth.

**Figure 5:**
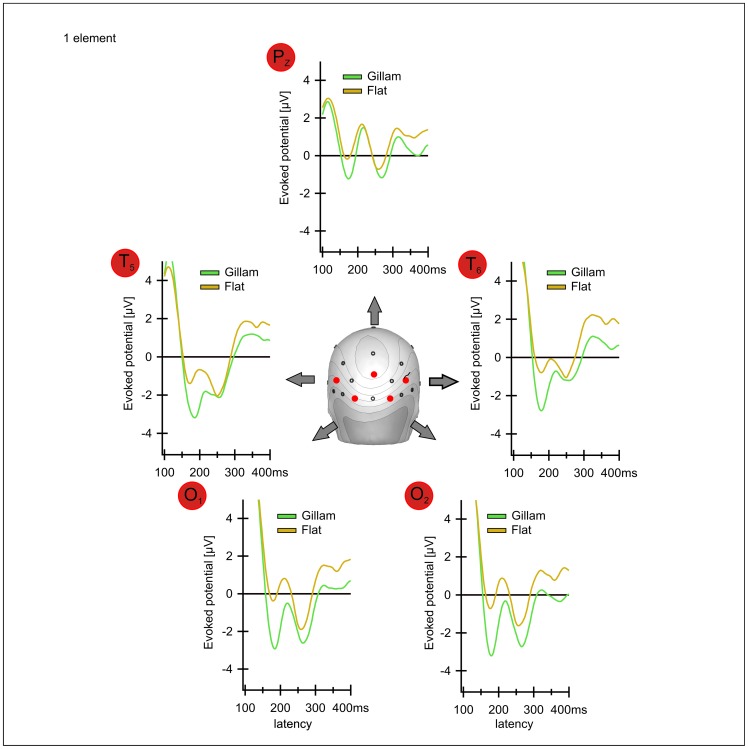
Grand averages evoked by “Gillam Stereo” (condition II, green lines) and the flat stimulus condition (VI, yellow lines) for all 5 electrode positions employed. The positions of the electrodes are marked by red dots on the schematics of the head in the center. Only the version with one element is shown, results for the version with 9 elements are similar. The smallest difference between the two conditions appears at electrode P_z_. The strongest difference is detectable at electrodes O_1_ and O_2_. Results for electrodes T_5_ and T_6_ are in between. Please note that the P270 of the Gillam stimulus is only traceable at electrodes O_1_ and O_2_.

Figure 5 plots the grand average-ERP curves between conditions II (“Gillam Stereo”) and VI (“Flat”) for all electrode sites to allow an overview over the distribution of the potentials specific for “Gillam Stereo” over the visual cortices. Inspection of this figure shows pronounced differences between flat and depth stimuli not just over the occipital pole but also temporally and parietally, although these differences were less pronounced than those over the occipital cortex.

**Figure 6:**
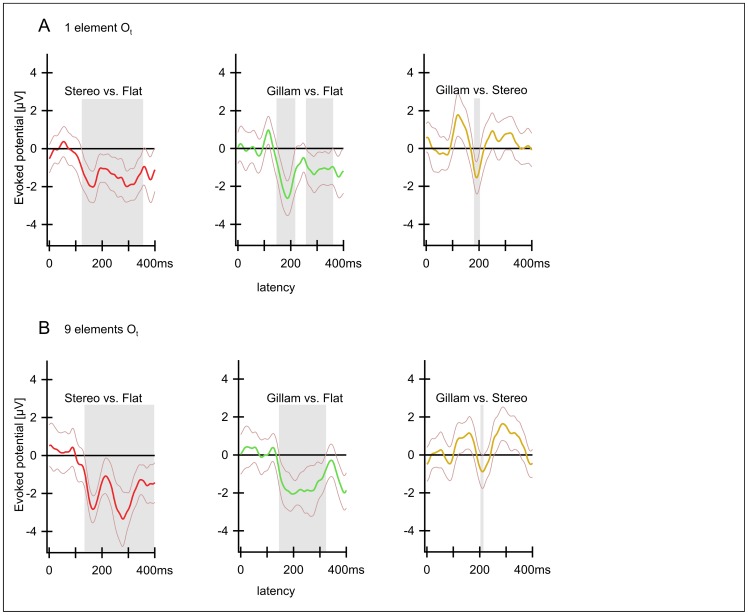
A, B Differences between the potentials evoked by Disparity Based Stereo, Gillam and Flat at electrode O_t_. A) Version with one element, B) Version with 9 elements. Solid lines indicate means of 12 observers, faint lines indicate confidence intervals (p = 0.05). Grey areas indicate the time intervals that differ significantly between the two conditions. Differences exist in extended time periods around P170 and P270. On the left the difference between Stereo (I) vs. Flat (VI) (red lines) is displayed. The results for these conditions differ between around 120 and 360 ms. The difference between Gillam (II) and Flat (VI) (middle, green lines) is significant over a similar time period. The difference between Gillam (II) and Stereo (I) (right yellow lines) is only significant at a latency of around 200 ms.

Figure 6A,B plots the **difference** potentials at electrode O_t_ between the cortical responses to “Stereo” (I) versus “Flat” (VI), “Gillam” (II) versus “Flat” as well as “Gillam” versus “Disparity Based Stereo” from left to right both for the one-element (A) and the nine-element stimuli (B) for better clarity. The thin lines above and below the potential curves indicate the confidence intervals of the means of all twelve subjects. Time spans where these confidence intervals are completely below zero differ significantly from zero. Hence the difference between the underlying conditions is significant during these time spans. These “significant” time spans are highlighted in grey. For both the one-element and the nine-element conditions the confidence intervals for the differences between the flat and the two stereo conditions are significant according to this definition. The distance between the line and the confidence intervals tends to be somewhat more pronounced in the nine element condition than in the one element condition, at least for the “Stereo” versus “Flat” condition.

The difference between the Gillam and stereo conditions produces results with confidence intervals that do not include zero for the N170, indicating a significant difference and possibly reflecting a greater mismatch in the Gillam case (an unmatched monocular contour). The N270, on the other hand, seems to be more pronounced in the stereo condition, at least for 9 elements, as is reflected in potential differences that deviate from zero in a **positive direction** (see Fig. 6B rightmost part; also the P100, not discussed in this manuscript, deviates significantly in positive direction from the Gillam versus Stereo condition due to a slightly larger amplitude and longer latency of the P100 in the Gillam condition, especially with 9 elements).

**Figure 7:**
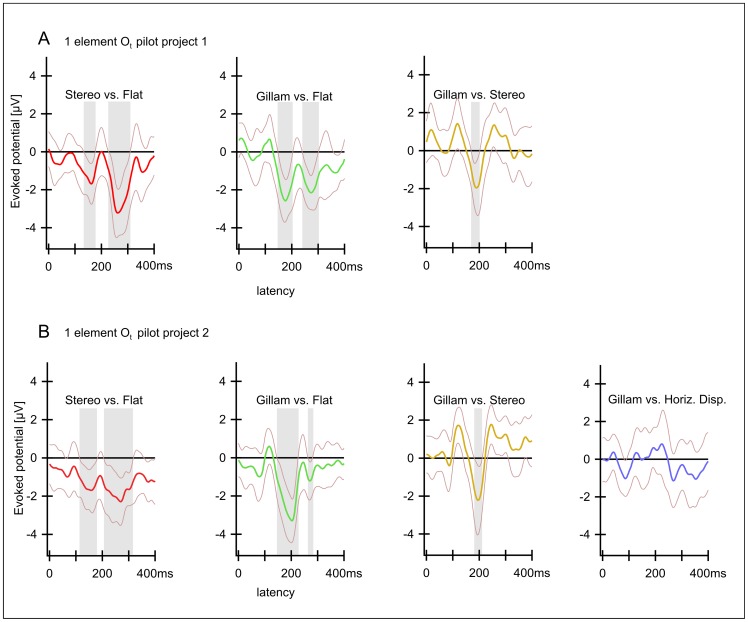
A, B Results of two pilot experiments with 7 observers (prior to the main study). Differences between potentials (compare Figs. 6 and 8). The two pilot experiments contained the conditions “Stereo”, “Gillam” and “Flat”, identical to the main study. But in the pilot project 1 (A) additional Stereo and Gillam conditions with “smaller disparities were tested (results not shown). In the pilot project 2 (B) the same conditions as in the main study were employed except Horizontal No Disparity”. Only the version with one element is shown (results for version with 9 elements were similar). Differences from left to right: “Stereo” (I) vs. “Flat” (VI); “Gillam” (II) vs. “Flat” (VI); “Gillam” (I) vs. “Stereo” (II); “Gillam” (II) vs. “Horizontal Disparity” (IV). Although these studies were conducted with a different set of observers and mixed with conditions differing from the main study the essential results are the same. There are two time intervals around the latencies of 170 ms and 270 ms where the conditions containing depth (“Stereo” I and “Gillam” II) differ from the flat condition (VI).

As outlined in detail in the Discussion our results indicate that indeed, Disparity Based and Gillam-type depth perception produce similar evoked potentials at short latencies that are most prominent over the occipital cortex. This notion is further supported by the very similar results of a pilot study conducted on 7 different observers (Figure 7 A, B) prior to the main study. We conclude that both types of depth impression may involve similar cortical areas at similar short latencies. But before we jump to this conclusion two possible confounds have to be excluded. These were.

**Figure 8:**
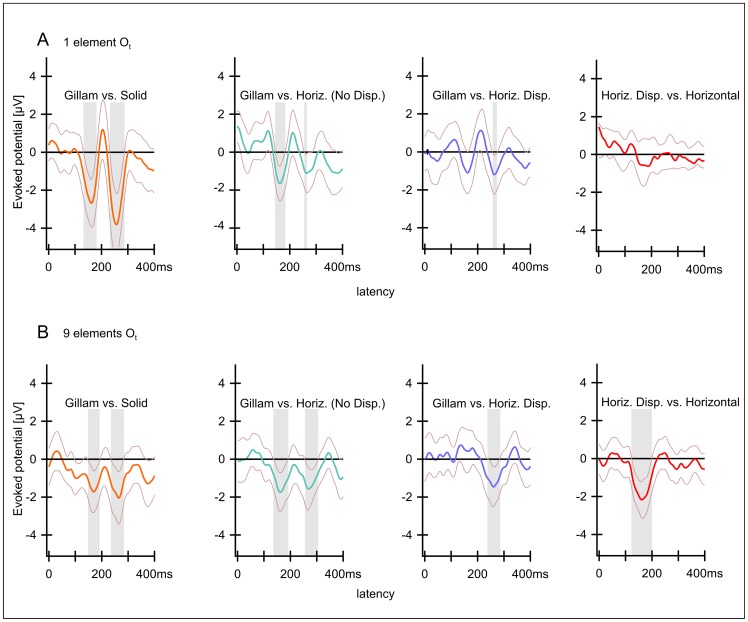
A, B Differences between potentials as in Figure 6, but between “Gillam”, “Solid”, “Horizontal” and “Horizontal Disparity”. A) Version with one element, B) Version with 9 elements. Differences from left to right: “Gillam” (II) vs. “Solid” (V); “Gillam” (II) vs. “Horizontal” (III); Gillam (II) vs. “Horizontal Disparity” (IV); “Horizontal Disparity” (IV) vs. “Horizontal” (III). Solid lines indicate means of 12 observers, faint lines indicate confidence intervals (p = 0.05). Grey areas indicate the time intervals that differ significantly between the two conditions. “Gillam Stereo” differs significantly from “Solid” both around 170 and 270 ms for the one and nine element conditions, and, to a lesser degree, from “Horizontal” (No Disparity). The difference between “Gillam” and “Horizontal Disparity” is significant at 270 ms, with a trend at 170 ms for the one element condition. Note that the Gillam stimulus is more similar to “Horizontal Disparity” than to “Horizontal” (No Disparity). The two Horizontal conditions differ only around 170 ms, and only for the nine element condition.

1. That the effect of “Gillam Stereo” is simply due to the fact that it has a non-fusible gap in the centre possibly resulting in rivalry (although this is not the experience one has with such a stimulus). This was controlled for by comparing evoked responses to the Gillam stimulus and the two stimuli with a *horizontal* gap in one eye, which should produce the same rivalry but not depth. A significant difference between the disparity based and Gillam stimuli on the one hand and the horizontal gap stimuli on other hand would make it unlikely that the presence of a non-fusible gap *per se* was responsible for the electrophysiological Gillam effect.
2. That the similarity of the results for “Disparity Based” and “Gillam Stereo” could be accounted for by the disparity at the outer edges of the rectangles in both cases. This seemed unlikely since (a) the Gillam condition produces a clear impressions of depth even without disparity at the outer edges although somewhat less clearly [13] and (b) as mentioned earlier neither depth threshold nor cross adaptation for Gillam stimuli resembled those for the same rectangles with the same edge disparity but no central monocular gap [14]. Nevertheless we included a solid rectangle with the same edge disparities as the disparity based and Gillam stereo stimuli here for comparison of evoked responses, to rule out this possibility.

### First control: The influence of unfusable or rivalrous contours

To address the first concern, we had incorporated in our experiment a first control condition, that allowed us to separate the relative contributions of rivalrous and depth cues.

We tested whether or not a similar type of difference as the one found between the “Gillam Stereo” stimulus (II) and the “Flat” stimulus emerges from a potentially rivalrous monocular contour that does not evoke a depth impression. Figure 8 shows the difference between “Gillam Stereo” and two conditions that both include such a cue conflict, namely, a monocular gap, but that does not lead to an impression of depth (“Horizontal Gap No Disparity” (III), “Horizontal Gap Disparity” (IV)). The “Horizontal Disparity” condition (III) differs from the Gillam condition only in the orientation of the gap. It yields significant differences from the Gillam stimulus in all conditions (only a non significant trend for 9 elements for the N270 peak). As to be expected, results for the “Horizontal No Disparity” condition (IV) also show a significant difference from the Gillam stimulus in all conditions – since here, the difference to the Gillam stimulus includes in addition the outer edges. These results indicate that “Gillam Stereo” is not merely a matter of rivalry. The orientation of the monocular gap matters.

### Second control: the influence of depth cues at the outer edges

To make sure that the disparities present in all our stimuli at the outer edges of all the rectangles presented are not the cause of the similarities between the disparity-based and the Gillam based stimuli and hence to address the second concern, we compared potentials between “Gillam Stereo” and a solid rectangle with no gap in either eye but with binocular disparities at the outer edges. (“Solid”, V). The potentials evoked by the Gillam condition differ significantly from those in the solid condition (Fig. 8, Table 3) (as they did from the “Flat” condition, see Fig. 6), even though the solid stimulus contains the only disparities present for the “Gillam” condition – those at the outer edges. Here, the only difference between the two stimuli compared consists in the monocular vertical gap only present in the Gillam condition. The different electrophysiological response for the “Solid” and “Gillam” condition affirms the difference obtained by Pianta and Gillam [14] indicating strongly that “Gillam Stereo” is not just a matter of transporting a depth signal from the edges of the figure to the gap. Another argument for the lack of importance of these outer edges for depth perception is that the N 270 does not differ between conditions horizontal disparity and horizontal no disparity, as mentioned above. (The N 170 may (nine element stimulus) or may not differ (one element stimulus)).

**Figure 9:**
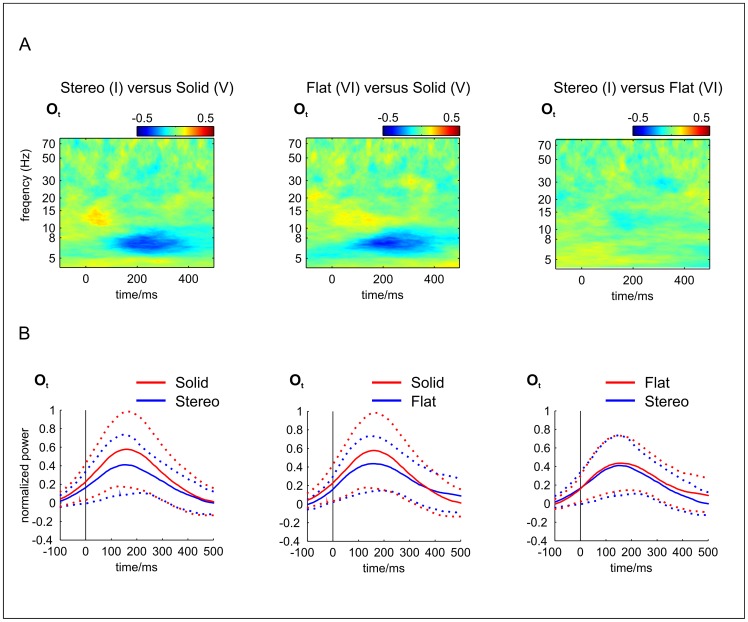
A, B Selected results of the wavelet – analysis. A) Time-frequency plots of **differences** in the power spectrum between the potentials evoked by conditions a) “Stereo” (II) vs. “Solid” (V), b) “Flat” (VI) vs. “Solid” (V) and c) “Stereo” vs. “Flat”, from left to right at electrode O_t_ in the group analysis (n = 12, only results of the version with one element are shown). Time indicates latency after stimulus presentation; frequency indicates the Fourier frequency analyzed. Colours code for normalized power (compare Material and Methods). Differences show up only in the slow wave band with the condition “Solid” (V) yielding most power. B) Averaged power amplitudes of the same contrasts between conditions as in A but only for the *θ*- band (4–8 Hz) as a function of time (latency). Dotted lines indicate standard errors of the corresponding mean. Statistically significant differences were only found for the contrasts of “Stereo” vs. “Solid” and “Flat” vs. “Solid”. This fact probably reflects the difference between the neural activity of presenting a rectangle containing a white gap versus no gap. As there is no difference between “Stereo” vs. “Flat” (compare figure on the right), the difference probably has nothing to do with the perception of depth as such.

In addition, we analysed the data using wavelet analysis, concentrating only on the theta band (see Materials and Methods, time frequency analysis). Statistically significant differences were only found for the contrasts of the “Solid Rectangle” versus the “Flat” condition (version with one element and with nine elements) and for the “Stereo” versus the “Solid Rectangle” condition (version one element only) (Fig. 9).

## Discussion

We will first summarize the main results and then discuss each of them in more detail. The results for stimuli with nine rather than one stimulus element are quite similar to those for one stimulus (Figs. 3, 4) and hence, these conditions will generally be discussed together. The most important question was whether or not stimuli containing Gillam depth features evoke cortical potentials similar to stimuli producing a stereoscopic impression based on disparity differences. An earlier study compared the cortical potentials evoked by a depth-defined checkerboard with those evoked by a homogeneous, i.e. planar version of the same stimulus) [19]. The difference between the cortical responses to these two stimuli hovered around zero for the first 100 ms after stimulus presentation and started to deviate towards more negative potentials for the checkerboard stimulus at around 170 ms. The amplitude of this negative potential increased over time, peaking at around 270 ms after stimulus onset. We therefore expected to find a similar potential also in the present study for those stimuli containing gradients in depth, and with a (negative) peak around 270 ms after stimulus presentation.

Figure 3 shows potentials evoked by “Disparity Based Stereo”, “Gillam Stereo” and a flat stimulus and Figure 6 shows all possible differences between these three conditions. Both depth stimuli produce larger N170 and N270 than the flat stimulus. As indicated above, very similar results were obtained for a stimulus containing nine (identical) elements and stimuli containing a single one (Figs. 3, 4, 6, 8 below). A pilot study on another 7 observers (Fig. 7) yielded similar results with similar latencies and with significant differences in confidence intervals.

Let us first consider the negativity at a latency of 270 ms. “Gillam Stereo” evokes cortical potentials that differ only slightly from those evoked by Disparity Based Stereo but which are significantly different from those evoked by stimuli that appear flat (Figs. 3, 6 and Table 1,2). To better visualize the difference between a flat rectangle (V) and one with a disparity gap (I), Fig. 6A, B (left and middle) plots this difference for both disparity and Gillam-based depth. This difference is highly significant (see Table 1, 2), indicating that a slight disparity can have a huge influence on the N270. This negative potential is lacking for stimuli not producing a clear impression of depth (Fig. 4), and may even reverse direction in the case of the “Solid Rectangle” (Fig. 4). Hence, we interpret this negativity as being related to the subjective impression of “depth”.

Less clear is the role of the negativity with a latency around 170 ms in our stimuli. The visual N170 is usually related to processing effort, attention or Gestalt perception and may involve stimuli as varied as Kanisza squares [24] or faces [25]. The N170 in our study is relatively similar for all stimuli containing stimuli involving non-corresponding retinal positions –either due to disparities and/or to non fusible line segments. It is absent only for the stimulus containing completely identical elements in both eyes, the flat rectangle with a vertical gap in both eyes (IV). One may speculate that this negativity signals that the stimuli to both eyes differ. It is surprising that even a slight disparity at the rectangles` outer border (that does not produce an impression of depth) suffices to trigger this early potential. It seems as if the visual system does detect the disparities at these outer borders but does not use them to compute depth.

A second point, demonstrated by the data from Fig. 4 and reanalysed in Fig. 8 right side is the fact that results were relatively independent of the presence or absence of disparities at the outer edges of the rectangle. The two conditions containing horizontal gaps (IV, VI) differ only in respect to this disparity of the outer edges of the rectangle: one contained such a difference while the other did not. The results for the two conditions are very similar for the ‘depth-component’ of the evoked response, at 270 ms whereas there is a significant difference for the earlier component N170 for the 9 element condition. This may be due to the greater difference between the monocular images for the horizontal disparity condition.

Wavelet analysis and subsequent calculation of differences for the conditions contrasted in Figs. 3, 4,6,8 leads to significant differences only in slow-wave band (Theta) between conditions “Stereo”, “Solid” and “Flat” (see Fig. 9). “Stereo” produces stronger activations in this frequency band than the “Solid” condition and the same is true for the “Flat” condition. This may be a consequence of the vertical gap in the middle of the rectangles.

In summary, we found that depth perception produced by the presence of a monocular gap in the binocular configuration we have called “Gillam Stereo” evokes cortical potentials very similar to those of “Disparity Based Stereo”, both in amplitude and latency, being most pronounced over occipital cortex. This we consider as an indication that the Gillam type of depth perception involves similar mechanisms and cortical areas as “Disparity Based Stereo” vision does, rather than being based on complex cognitive processes that may require longer processing times and involve more “upstream” cortical areas. We conjecture that (as in the case of “subjective contours” [26], rather local and “low-level” mechanisms may produce a depth percept, based on unmatched vertical contours, while a top-down influence of “upstream” areas, of course, may be involved given a latency of 270 ms. In addition, we found clear evidence that unmatched contours in the two eyes evoke relatively early potentials even if these contours can be resolved (in a second step?) to produce stereoscopic depth. These results indicate a two-step processing of contours that do not fall on (exactly) corresponding parts of both retinas. In a first step, the visual cortex reacts differentially to contours that are projected on to not exactly corresponding parts of both retinas. This mismatch of contours produces or at least contributes to a negativity around 170 ms after stimulus onset. In a second step, these contours either elicit the impression of depth based on fusion or on the presence of an ecologically valid monocular contour. This step produces a negativity with a latency around 270 ms. It remains to be clarified how the cortex is able to achieve this fast 3-D interpretation in the case of “Gillam Stereo”.
